# In the wake of the Scandinavian Simvastatin Survival Study trial

**DOI:** 10.1097/MOL.0000000000001008

**Published:** 2025-08-12

**Authors:** Timo E. Strandberg

**Affiliations:** a Department of Medicine, University of Helsinki and Helsinki University Hospital, Helsinki; b University of Oulu, Center for Life Course Health Research, Oulu, Finland

**Keywords:** adherence, atherosclerotic cardiovascular disease, dyslipidaemia, LDL-cholesterol, statin

## Abstract

**Purpose of review:**

In 1994, the 4S trial was revolutionary by showing that cholesterol lowering with simvastatin reduced, not only atherosclerotic vascular disease (ASCVD) events, but also all-cause mortality as compared to placebo. During the following 30 years, statins have proved to be well tolerated and effective and also paved way for new innovations in the field of dyslipidaemia therapy.

**Recent findings:**

The aim of this review is to summarize current knowledge about statins and effects of cholesterol-lowering accumulated in the wake of 4S trial: both vascular and nonvascular benefits, adverse effects, adherence, and statin intolerance. While secondary prevention of ASCVD has emphasized ‘the lower the better’ in LDL-cholesterol lowering, emerging topic is ‘the longer the better’ to reduce lifetime LDL burden and achieve full potential of ASCVD prevention. With statins as backbone therapy, new treatment innovations are in trials to better manage all atherosclerotic lipoproteins and residual risk.

**Summary:**

After becoming generic, statins are inexpensive and well tolerated therapy with potential to substantially reduce the burden of atherosclerotic vascular disease world-wide. To achieve these goals, both accessibility and adherence are fundamental issues.

## INTRODUCTION

In medicine, it often takes 20–30 years before important discoveries are turned into clinical practice. That was the case with inhibitors of 3-hydroxy-3-methylglutaryl coenzyme A ((HMG-CoA) reductase (‘statins’), too. Over 20 years after Akira Endo had presented his discoveries about the effects of the first inhibitor of cholesterol synthesis (mevastatin) on lowering serum cholesterol, the first clinical endpoint study – Scandinavian Simvastatin Survival Study (4S) – showed its remarkable results in the prevention of atherosclerotic cardiovascular disease (ASCVD, [1,2^▪^]). Participants of 4S were at high risk – all had coronary artery disease and mean baseline LDL-cholesterol (LDL-C) was quite high, 4.9 mmol/l. On the other hand, compared to current standards, simvastatin dose was low, mean 27 mg/day. Importantly, also all-cause mortality was reduced 30% in the group receiving simvastatin for 5.4 years as compared to the placebo group, thus abolishing fears of increasing non-CVD events and serious adverse effects during statin treatment (Table 1). Posttrial follow-up of 4S showed that simvastatin benefit prevailed and even increased [3]. In all, 4S results have been instrumental to prove the paradigm of LDL-C, reflecting LDL particles and apolipoprotein B (apoB), as the primary cause of ASCVD [4,5]. 

**Box 1:**
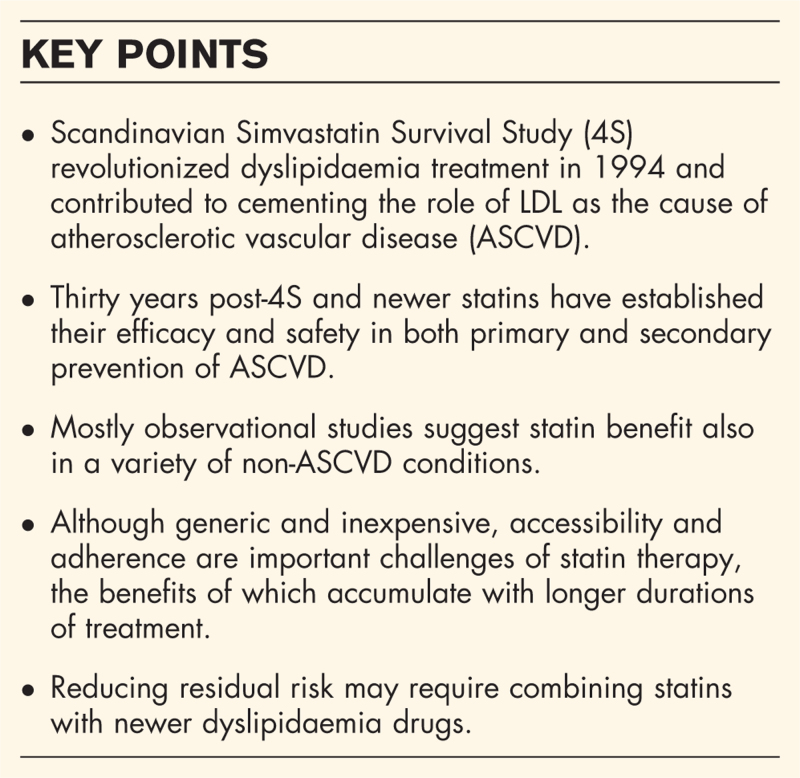
no caption available

**Table 1:** Summary results of the Scandinavian Simvastatin Survival Study

	Placebo (*n* = 2223)	Simvastatin 20–40 mg/day (*n* = 2221)	Relative risk (95% confidence interval)
Baseline LDL-cholesterol	Mean 4.9 mmol/l (190 mg/dl)	Mean 4.9 mmol/l (190 mg/dl)	
Change of LDL-cholesterol during the study	Mean 1% increase	Mean 35% decrease	
Fatal events (%)			
All-cause	11.5	8.2	0.70 (0.58–0.85) Significant in men, nonsignificant in women
Coronary	8.5	5.0	0.58 (0.46–0.73) Significant in both sexes
All cardiovascular	9.3	6.1	0.65 (0.52–0.80)
All noncardiovascular	2.2	2.1	..
Nonfatal events (%)			
Any major coronary	22.6	15.9	
Any cerebrovascular	4.3	2.7	
Cancer	2.7	2.6	
Discontinuation due to adverse effect (%)	6	6	

In the Scandinavian Simvastatin Survival Study (4S), 4444 people (mean age 58 years, 81% men) with coronary heart disease (79% with history of myocardial infarction) and serum cholesterol level 5.5–8.0 mmol/l were randomized into a placebo group and a simvastatin group for a median of 5.4 years. Primary endpoint was total mortality.

4S can be seen as a turning point and stimulus for dyslipidaemia treatment in general. Although both observational and randomized diet studies during the 1950s–1960s had suggested benefit of cholesterol lowering and led to several trials with hypolipidaemic drugs, the field was quite controversial at the beginning of 1990s [4]. Trials with cholestyramine and gemfibrozil were successful in preventing coronary endpoints, but in primary prevention trials, the absolute benefits were not large, and serious adverse effects especially with clofibrate aroused concern [4]. Some scholars considered cholesterol as a vital substance for life, and inhibiting cholesterol synthesis was deemed potentially dangerous [6,7]. In addition to adverse muscle effects, statins were feared to cause cancer, cataracts, liver toxicity, cognitive disorders and damage to heart muscle to name a few. Although 4S and thereafter numerous studies have proved these fears mostly to be false, seeds of old fears may still haunt many minds.

First statins were natural (lovastatin, pravastatin) or semi-synthetic (simvastatin), but more efficient synthetic statins (atorvastatin, rosuvastatin) followed, and by 2008, major statin trials both in primary and secondary prevention had replicated over and over again statin efficacy and safety [8^▪^,^9^–^11^,12^▪^]. It is of note that after early 2000s, comparison of statin to placebo has been largely considered to be ethical only in special subgroups; therefore, the efficacy of statins in comparison to control treatment seems diluted. After patents have gradually expired, all statins except pitavastatin are now generic and consequently very inexpensive therapy, with hundreds of million users world-wide. Still, ‘the cholesterol wars’ [4] are not finally over, and statins remain underused [2^▪^] with insufficient adherence and their use may even be disturbed by frank ‘statin denial’ [13]. At least partly these are due to scepticism about cholesterol as a cardiovascular risk factor and experience from prestatin hypocholesterolaemic drugs [4].

In the following review, the aim is to summarize current knowledge accumulated in the wake of 4S about statin benefits – vascular and nonvascular – as well as their adverse effects and statin intolerance. I also consider the current status of statins among newer dyslipidaemia drugs to reach LDL-C targets and potential ways to improve statin adherence. The latter is crucial for obtaining full benefit of this therapy potentially capable to curb the global epidemic of ASCVD.

## CARDIOVASCULAR BENEFITS OF STATINS

During the 30 years after the 4S trial, the clinical benefits of statins in the prevention of ASCVD are well established, and statins are certainly one of the best documented drug therapies. Numerous systematic reviews and meta-analyses of randomized controlled trials (RCTs) have been presented, and statins have shown consistent benefit in both primary and secondary prevention and in various subgroups [8^▪^,^9^–^11^,12^▪^]. In trials with usual duration for 2–5 years, 1 mmol/l reduction of LDL-C has conferred a 22% reduction of ASCVD risk, and relative benefit appears to sustain in secondary prevention although may be diluted in primary prevention in older age [12^▪^]. This is probably due to the late initiation when subclinical ASCVD has already progressed in the usual age of participants between 55 and 70 years. Ongoing placebo-controlled primary prevention RCTs will shed more information about starting statin treatment in older people, more than 70 years of age [14,15].

As a whole, experience from statin RCTs, observational and Mendelian randomisation studies support both the ‘lower the better’ and the ‘longer the better’ paradigms in the prevention of ASCVD. In the primary prevention trial, benefit from pravastatin continued to increase during 20 years’ follow-up [16], and a recent detailed analysis of RCTs also suggested that the benefits of LDL-C lowering are not fixed but increase steadily with longer durations of treatment [17]. Accordingly, each millimole per litre lower LDL-C may actually be associated with more than 50% lower coronary risk, when the duration of exposure is taken into account.

With increasing evidence of safety, statin treatment has even been extended to children, from age 6 years, and adolescents with familial hypercholesterolaemia (FH) [18,19^▪^].

## PLEIOTROPIC EFFECTS: FACT OR FICTION?

Cholesterol is not the only end product of HMG-CoA, and in that sense, inhibition of HMG-CoA reductase has a potential for pleiotropic effects, that is, not related to effects via upregulating LDL receptor [20]. These pleiotropic effects are usually considered to include anti-inflammatory, antihypertrophic, antifibrotic, and antioxidant effects on platelets, endothelial function, and components of the vascular wall. They have been demonstrated in preclinical studies, but not consistently translated in large RCTs, and it is also unclear to what extent these are in the end due to lipid effects of statins. It is of note that LDL-C reduction provides equal benefit in ASCVD prevention, independent from the mechanisms by which LDL-C are lowered (eg [ PCSK9] inhibitors, bembedoic acid). In fact, whether statins have clinically meaningful pleiotropic effects has been debated since the introduction of statins, and final conclusions are still pending [20]. While it is entirely possible that meaningful pleiotropic effects of statins are not fully achieved with clinically safe doses, various effects of statins in non-ASCVD conditions – as listed in the next chapter – suggest also effects not directly related to cholesterol lowering.

## BENEFITS IN NON-ATHEROSCLEROTIC CARDIOVASCULAR DISEASE CONDITIONS

Patients with any disease, but with comorbid risk of ASCVD like diabetes and chronic inflammatory disease, may benefit from statin treatment through prevention of ASCVD-related outcomes [21^▪▪^]. However, there is also mainly observational and some RCT evidence that statins would benefit non-ASCVDs, possibly via pleiotropic effects. On the other hand, cholesterol toxicity may be involved in various non-ASCVD conditions [22]. Exact mechanisms independent of lipid effects are difficult to study, though, and specific placebo-controlled RCTs are increasingly challenging to organize because of prevalent statin use. Not being a systematic review, some non-ASCVD diseases or conditions where statin benefits have been reported are shown in Table 2[21^▪▪^,^23^,24^▪^,^25^–^29^,^30^^▪▪^,^31^–^33^,34^▪^,^35^–^39^,^40^^▪▪^,^41^–^44^, 45^▪^,^46^].

**Table 2:** Effects of statins on some nonatherosclerotic cardiovascular disease conditions and risk factors

Condition	Notes	References
Diabetes	Reduction of ASCVD events in several RCTs.	[24^▪^]
Chronic kidney disease	Statins lower the risk of ASCVD comorbidity, but no clinically meaningful effect on kidney outcomes.	[25,26]
Heart failure	Statins prevent development of heart failure via prevent of CAD; prevent ASCVD comorbidity; may prevent progression of heart failure but have no benefit in end-stage disease.	[27,28]
Venous thromboembolism (VTE)	Statins for the primary prevention of VTE may slightly reduce the incidence of VTE and all-cause mortality.	[29]
Metabolic dysfunction-associated steatotic liver disease (MASLD), metabolic dysfunction-associated steatohepatitis (MASH)	Improvement of biochemical risk factors of ASCVD; possibility to prevent complications like cirrhosis and liver cancer.	[21^▪▪^,^30^^▪▪^,^31^]
Immune–mediated inflammatory conditions	Patients are at increased risk of ASCVD, which can be reduced with statins.	[21^▪▪^]
Infections	Improved prognosis in many observational studies but no RCT evidence; chronic use may protect the host by reducing risk of ASCVD complications, for example, in HIV.	[32,33,34^▪^,^35^]
Cancer	Inconsistent results in various cancer types and no specific RCTs. However, potential benefit has been reported at least for liver, prostate, breast, and colon cancer. Reduction of ASCVD complications in cancer patients is a plausible effect.	[30^▪▪^,^35^–^37^]
Osteoporosis and fractures	Potential for decreased risk of fractures and increased bone mass density.	[38]
Cognition/dementia	There is no evidence of cognitive impairment during statin treatment in observational studies nor in RCTs. Potential effect through prevention of ischemic strokes; prevention of ASCVD complications in patients with cognitive disorders; futile in secondary prevention of Alzheimer patients; primary prevention trials are ongoing.	[39,40^▪▪^,^41^]
Idiopathic pulmonary fibrosis	Inconsistent benefit, although antifibrotic effect potentially favourable.	[42]
Frailty	No evidence of adverse effects in observational studies and potential benefit through prevention of ASCVD comorbidity.	[43,44,45^▪^]
Organ transplants	Patients are at increased risk of ASCVD, which can be reduced with statins.	[21^▪▪^]
Subarachnoid haemorrhage	Potential benefit especially in patients with hypertension and cerebrovascular disease.	[46]

ASCVD, atherosclerotic cardiovascular disease; RCT, randomized controlled trial.

The benefit may also depend on timing: benefit of statins may exist in earlier but not in late stages of the disease. This applies, for example, to chronic kidney disease, heart failure, cognitive disorders, and possibly frailty (Table 2).

## ADVERSE EFFECTS

Already at the time of 4S, it was known that statin treatment can affect muscles, liver and possibly eyes (cataract), and participants were carefully monitored for adverse effects in those organs [1]. Creatine phosphokinase (CK) and liver transaminases were measured regularly and ophthalmologic examinations were performed annually during 4S. However, serious adverse effects in the placebo-controlled trial were very rare, and it is remarkable that discontinuation rate due to adverse effects was only 6% during 5 years reflecting that nocebo effect was not yet operating! Large post-4S trials also with more powerful statins have largely corroborated the early experience with the exception of increased diabetes risk, which was first reported in the JUPITER trial with rosuvastatin in 2008 [47].

It is very important to discern real adverse effects and true statin intolerance. Adverse effects on muscle, liver, kidney, and glucose metabolism are discussed in the following.

### Muscle effects

Incidence of muscle symptoms has been quite high in observational statin studies, but a nocebo reaction is common as verified by placebo-controlled studies [48^▪^]. In RCTs, statin therapy has caused a small excess of usually mild muscle pain, and over 90% of all reports of muscle symptoms were not due to the statin [49]. Consequently, routine examination of CK is not needed, but taking patient complaints seriously is important also for adherence reasons. Skeletal muscle adverse effects are possible with statins as shown with drugs interactions or with certain genetic effects leading to very high statin levels in blood [50]. Mechanisms are probably multifactorial [51]. Very rare idiosyncratic reactions, like immune necrotizing myopathy, have been reported during statin therapy, but causality is not established [52^▪^].

There has been discussion whether statin effects on skeletal muscle also apply to cardiomyocytes [53]. Proven cardioprotective effects of statins obviously counteract this, and muscle types differ biologically [53]. Although rosuvastatin treatment had limited benefit in patients with systolic heart failure in the CORONA trial, excessive adverse episodes were not observed [54].

### Hepatic effects

Despite the liver being the target organ of statins, decades of use have proved hepatic safety – increasing evidence rather suggest hepatoprotective effects (Table 2). Increase of transaminases may occur but have probably no clinical significance and do not necessitate discontinuation [8^▪^,^9^]. Excess elevation of liver enzymes (e.g. more than three times upper limit) naturally require appropriate examinations for specific liver disease. Routine monitoring of transaminases is not necessary.

### Kidney effects

High-potency statins may promote proteinuria, but clinical significance is unclear, and statins are the cornerstone of therapy in early-to-moderate chronic kidney disease [24^▪^,^25^]. However, statin efficacy diminishes in advanced stages of kidney disease.

### Diabetes risk

After the first report of diabetes risk associated with statin therapy in 2008 [47], several studies have examined the relationship. According to a meta-analysis of randomized trials, statins cause a dose-dependent and moderate increase of diabetes incidence [55^▪▪^]. However, the majority of cases occur among individuals already at risk of diabetes before statin therapy. This emphasizes the importance of weight control and healthy diet also during statin treatment.

Of note, in the West of Scotland Coronary Prevention Study (WOSCOPS), development of type 2 diabetes mellitus was found to decrease by 30% in pravastatin-treated patients [56]. It was suggested that anti-inflammatory and endothelial effects of pravastatin could be responsible as well as its lipid-lowering effect. Consequently, the association of statin and diabetes mellitus may not be straightforward.

### Older patients with frailty

There is no evidence that statin therapy would predispose to frailty [57], but concerns about statins in old, frail individuals are nevertheless common. These concerns are mainly based on multimorbidity and polypharmacy and potential mobility problems caused by statin effects on muscle. At the moment, there is no reason to discontinue treatment after 75 years of age nor refraining start of treatment in secondary prevention [58^▪▪^]. Ongoing trials comparing atorvastatin and placebo among 70–75+ individuals will cast more light on the efficacy in primary prevention during coming years [14,15]. At end of life and with terminal illness, discontinuation of preventive treatments like statins is relevant, but otherwise, deprescribing statin therapy is not straightforward and may cause harm [59,60].

### Miscellaneous effects

Since the start of their clinical use, a myriad of adverse effects have been suspected to associate with statin therapy, and they have also been listed in product information. These include various gastroenterological, neurological, immunological, and metabolic effects. With exceptions listed above, they have not been verified in RCTs, and for some, even the contrary may be true. As unfounded information about adverse effects is apt to reduce adherence to statin therapy, accurate and truthful reporting of adverse effects is of great importance.

## STATIN INTOLERANCE

In general, statin intolerance has been defined as one or more adverse effects – most commonly muscle effects – during statin therapy, which resolve or improve with dose reduction or discontinuation [48^▪^,^61^,^62^]. Statin intolerance can be complete or partial. To classify a patient as having statin intolerance, therapy with a minimum of two statins should have been tried, at least one at the lowest approved daily dosage. In contrast to blinded studies like 4S, statin intolerance may be reported in up to 30% of patients in observational studies, hinting for nondrug, nonspecific effects. Based on blinded trials an important reason for ‘statin intolerance’ is the nocebo effect, whereby the patient expects harm resulting in perceived adverse effects.

Perceived statin intolerance nevertheless contributes to reduced persistence and adherence with ensuing higher risk for adverse ASCVD outcomes. Consequently, various ways have been developed to accurately define true statin intolerance to help clinicians. Several different strategies can be used to identify a tolerable statin regimen. These may include using different statins, dose, dosing frequency, and combination therapy to reduce statin dose [48^▪^]. It is very important to take the patient's concerns seriously, inform about the potential nocebo effect and discuss for possible solutions. In the end, nonstatin therapy with ezetimibe, bembedoic acid, or proprotein convertase subtilisin/kexin type 9 (PCSK9) inhibitor may be required.

## HOW TO IMPROVE GOAL ATTAINMENT AND ADHERENCE

With accumulating evidence, targets especially in secondary prevention have become lower, an example the current ESC/ESH recommendation of LDL-C less than 1.4 mmol/l [63]. A frequent concern is how statin treatment can cope with this and to what extent substantially more expensive drugs like PCSK9 inhibitors are needed. Even the most powerful statin may be insufficient, but combination with generic ezetimibe markedly improves goal attainment. According to recent European surveys (EUROASPIREV and SANTORINI), targets are poorly met in clinical practice [64,65]. In addition to ezetimibe, statins can combined with plant stanol/sterol margarine, bempedoic acid, and PCSK9 inhibitors to better reach recommended goals [2^▪^].

Nonadherence is a serious problem, and forgoing evidence-based treatments may also cause substantial economic loss [66]. Importantly, it is long known that discontinuation of statin therapy predisposes to unnecessary ASCVD events [67]. Several ways to improve statin adherence have been presented [2^▪^,^68^]. In general, most important is to give truthful and sufficient information about not only statin benefits but also risks, and take patient complaints and concerns about adverse effects seriously. In the end, true statin intolerance is less frequent than commonly believed. That was implicated already in the 4S trial.

## CONCLUSION

Considering the scepticism of the cholesterol theory 30 years ago [4], one may well argue that the fate, or at least progress, of dyslipidaemia treatment hinged on 4S results. Therefore, 4S was instrumental for strengthening the role of plasma LDL level as a *sine qua non* in developing ASCVD in the first place [5]. Gradually, the importance of cumulative exposure to LDL-C, the so-called ‘cholesterol life-years’, has been underscored by observational and genetic evidence, and it is now fundamental for designing preventive strategies. An important notion is that the benefits of LDL-C lowering do not seem to be fixed but increase steadily with longer durations of treatment (‘the cumulative exposure hypothesis’ [17,69^▪▪^]). Although total ASCVD risk has been the metrics to guide statin therapy for primary prevention in many guidelines, it is worth noticing that it was LDL-C level, which guided participant inclusion in statin RCTs showing benefit [70^▪▪^,^71^^▪▪^]. This emphasizes primordial prevention, that is, focus on the young to prevent atherosclerotic risk factors – not only dyslipidaemia – from developing in the first place, and the need to recognize elevated lipid levels in genetic conditions like FH or high lipoprotein(a) as early as possible. Because statins are well tolerated and inexpensive and because age reflects CVD burden, suggested age-based and LDL-C-based treatment would be ideal to control development of atherosclerosis [71^▪▪^]. This emphasizes challenges to resist misinformation about statins and improve accessibility to measurement and adherence to LDL-C reduction (Table 3) [72].

**Table 3:** Future global roadmap for better dyslipidaemia therapy

Risk assessment
The importance of life-course – primordial prevention
Focus on lifetime risk
Case finding
Affordable testing early in the disease course
Universal and early screening for genetic dyslipidaemias
Treatment
Wider availability of affordable, effective and cost-effective treatment regimens
Polypill approach
Adherence
Better health literacy to combat misinformation about treatments
Multidisciplinary teams and applications to support adherence and patient-centred therapy
Future trials
New approaches: Better use of pragmatic trials and trials with high-quality imaging to reduce costs
Equity in trials: sex balance, ethnic groups, also multimorbid and frail patients

Modified from ref. [72].

As treatment in real life is not usually started until the atherosclerotic process – clinical or subclinical – is advanced, the residual risk of ASCVD is substantial and requires effective control of not only LDL-C (with nonstatin medications as needed), but also other atherogenic lipoproteins and probably inflammation [2^▪^]. During the post-4S decades, obesity epidemic has increased world-wide and strengthened the role of remnant lipoproteins and inflammation in addition to LDL-C. Also the special characteristics of ApoB, ceramides, and effects beyond cholesterol are being elucidated [73^▪^]. Although are also partly benefitted by statin treatment, numerous novel therapies are being studied in “4S 2.0” trials [2^▪^]. and advanced imaging techniques of subclinical ASCVD to help better target therapy in primary prevention [74^▪▪^].

## Acknowledgements

*None*.

### Financial support and sponsorship

*Päivikki and Sakari Sohlberg Foundation; Helsinki University Hospital (VTR Funding).*

*No funding received for this work from the following organizations: National Institutes of Health (NIH); Wellcome Trust; and Howard Hughes Medical Institute (HHMI).*

### Conflicts of interest

*Reports consultancies and talks sponsored by Amarin, Amgen, CoroPrevention, Duodecim, Finnish Medical Journal, GSK, MSD, Novartis, Nutricia, Orion Pharma, Sankyo, Valio. Chairperson of the national dyslipidemia guideline group.*

## REFERENCES AND RECOMMENDED READING

Papers of particular interest, published within the annual period of review, have been highlighted as:

▪ of special interest
▪▪ of outstanding interest
